# Non-vector-borne transmission of lumpy skin disease virus

**DOI:** 10.1038/s41598-020-64029-w

**Published:** 2020-05-04

**Authors:** Kononov Aleksandr, Byadovskaya Olga, Wallace B. David, Prutnikov Pavel, Pestova Yana, Kononova Svetlana, Nesterov Alexander, Rusaleev Vladimir, Lozovoy Dmitriy, Sprygin Alexander

**Affiliations:** 1 Federal Center for Animal Health, Vladimir, Russia; 2 Agricultural Research Council-Onderstepoort Veterinary Institute, P/Bag X5, 0110 Onderstepoort, South Africa; 3 Department Veterinary Tropical Diseases, Faculty of Veterinary Science, University of Pretoria, P/Bag X4, 0110 Onderstepoort, South Africa

**Keywords:** Biological techniques, Biological models, Animal disease models

## Abstract

The transmission of “lumpy skin disease virus” (LSDV) has prompted intensive research efforts due to the rapid spread and high impact of the disease in recent years, especially in Eastern Europe and Balkan countries. In this study, we experimentally evaluate the vaccine-derived virulent recombinant LSDV strain (Saratov/2017) and provide solid evidence on the capacity of the virus for transmission in a vector-proof environment. In the 60-day long experiment, we used inoculated bulls (IN group) and two groups of in-contact animals (C1 and C2), with the former (C1) being in contact with the inoculated animals at the onset of the trial and the latter (C2) being introduced at day 33 of the experiment. The infection in both groups of contact animals was confirmed clinically, serologically and virologically, and viremia was demonstrated in blood, nasal and ocular excretions, using molecular tools. Further studies into LSDV biology are a priority to gain insights into whether the hypothesized indirect contact mode evidenced in this study is a *de novo*-created feature, absent from both parental stains of the novel (recombinant) LSDV isolate used, or whether it was dormant, but then unlocked by the process of genetic recombination. Author summary: In global terms, LSD has been termed a “neglected disease” due to its historic natural occurrence of being restricted to Africa and, occasionally, Israel. However, after its slow spread throughout the Middle East, the disease is now experiencing a resurgence of research interest following a recent and rapid spread into more northern latitudes. Given the dearth of solid findings on potential transmission mechanisms, no efficient or reliable control program currently exists, which does not involve the use of live attenuated vaccines or stamping out policies – both of which are controversial for implementation in non-endemic regions or countries. The vector-borne mode is the only working concept currently available, but with scarce evidence to support the aggressive spread northwards – except for human-assisted spread, including legal or illegal animal transportation. The emergence of outbreaks is not consistently linked to weather conditions, with the potential for new outbreaks to occur and spread rapidly. Here, for the first time, we provide evidence for indirect contact-mode transmission for a naturally-occurring recombinant LSDV isolated from the field. In an insect-proof facility, we obtained solid evidence that the novel LSDV strain can pass to in-contact animals. Given the recombinant nature of the virus utilised, its genetic background relating to the observed transmission pattern within the study needs to be delineated.

## Introduction

Lumpy skin disease (LSD) virus belongs to the genus *Capripoxvirus* within the family *Poxviridae* (Buller *et al*., 2005). It contains a double-stranded, covalently-linked linear DNA genome enveloped by a lipid bilayer. Mature capripox virions have an oval profile and large lateral bodies, and they are 320 ×260 nm on average in size^1^. The viral genome is approximately 151 kb in size, encoding 156 putative genes^2^. LSD virus (LSDV) genes share a high degree of co-linearity and amino acid identity (average of 65%) with genes of other known mammalian poxviruses, particularly suipoxvirus, yatapoxvirus, and leporipoxvirus. Within the genus *Capripoxvirus*, which also includes sheep pox and goat pox viruses, genetic identities are at least 96%^3^. Generally, based on full-genome sequences deposited in GenBank, LSDV exists as two genetic lineages: field isolates and live attenuated vaccine strains, although evidence for a naturally occurring recombinant between a vaccine strain and field strain has also been reported^4^.

Displaying a strict and narrow host range, LSDV mainly infects cattle and water buffaloes^5^, with one documented isolation from sheep^6,7^, although a limited number of other ruminant species, such as antelope, also appear to be susceptible. In recent decades, the disease has been documented in both Southern and Northern hemispheres due to a rapid expansion of its range, although historically, it was restricted to the African continent^8–10^. The unprecedented incursions into new territories and resurgence of LSD creates an objective need for joint efforts to investigate the obscure nature of LSDV transmission^11^.

Initial evidence and studies suggested that direct or indirect contact (without vectors) are ineffective routes for LSDV transmission^12^, whereas the predominant transmission pathway for sheep pox and goat pox viruses is via aerosols. However, importantly, other poxviruses are most commonly spread by direct contact rather than by transmission by arthropod vectors such as by contaminated insects during biting of their hosts. Moreover, poxvirus infection can also occur through ingestion, parenteral inoculation, or droplet or aerosol exposure to mucous membranes or broken skin. Some poxviruses can be transmitted by fomites (inanimate objects). Transmission pathways may vary among poxviruses even within a genus^13^. For orthopoxviruses that infect humans, infection occurs via exposure to aerosols or droplets (variola virus) or through close personal contact. Poxviruses from the *Parapoxvirus* genus (e.g. orf virus or milker’s nodule virus) can pass from one animal to another through direct or indirect contact. Unfortunately, there are no available studies addressing the issue whether closely related capripoxviruses actually employ different routes of spread.

Transmission of LSDV is surmised to occur through mechanical vector-borne spread via insect or tick bites as most outbreaks occur during the warmer (and, often, wetter) summer months when potential vector species numbers are high^11,14,15^. Unfortunately, transmission studies conducted to date using arthropod species which may be involved in transmission are mostly species which are restricted to the Southern hemisphere (e.g. *Rhipicephalus appendiculatus* ticks), which complicates analysis of transmission potential of local species in climatically new geographic areas in the Northern hemisphere. Moreover, LSD outbreaks are not only reported within the warmer summer period, optimal for arthropod blood meal search activity, but also outside of it.This observation thus points to a possible non-vector-borne route for spread of the virus (WAHIS, 2019). Studies using a small cohort of animals were conducted in the past to show that direct contact transmission between infected and naïve animals is possible, but at an extremely low efficiency rate^12,16^. New work to replicate the findings has not yet been encouraged. Nevertheless, field evidence suggests that successful transmission can be achieved when naïve animals are allowed to share a drinking trough with severely infected animals^17,18^.

This supports the common hypothesis that direct contact does not appear to be an effective route for LSDV transmission. In addition, recent experiments with various field strains did not result in successful contact transmission^19–21^. Attenuated vaccine strains have also been claimed to be devoid of transmission capacity, but recent field evidence argues to the contrary^22,23^.

In this environment of uncertainty for LSD spread, elucidation of the exact transmission mode/s could contribute significantly towards improving control and eradication programs.

In this paper, we report on work performed to evaluate transmission of the naturally occurring vaccine-derived virulent recombinant strain of LSDV Saratov/2017^4^, in an experimental setting and for the first time conclusively demonstrate non-vector-borne transmission of the virus.

## Materials and methods

### Virus

The vaccine-derived virulent recombinant LSDV strain, LSDV Saratov/2017, was isolated by FGBI ARRIAH researchers from a cow presenting with severe clinical signs of LSD^22^. The strain was obtained from the FGBI ARRIAH depository and refreshed using two rounds of passaging in goat testis cells. To prepare the final inoculum virus, the refreshed virus was subjected to polymerase chain reaction (PCR) amplification of different loci of vaccine and field strain genomes to confirm the identity of this virus strain^4^.

### Ethics statement

The animal experiment, as well as the euthanasia procedure, were approved by the Ethics Committee of the Federal Center for Animal Health, Russia (Permit Number: №2/1-21082018) and conducted in strict accordance with Directive 2010/63/EU on the protection of animals used for scientific purposes.

### Experimental design

The initial experimental group consisted of 10 bulls of the Russian Black Pied breed aged 6-8 months. The animals were consecutively numbered from 1 to 10 in a random fashion and maintained in Animal Biosafety Level 3 housing with a 12-hourly light-dark cycle, relative humidity of 30% to 70%, temperature of 23 to 26 °C and all animals were monitored twice daily by the veterinary staff. Water and feed were provided *ad libitum*. The experiment was carried out in an insect-proof facility. To detect any possible dipteran presence, indoor blood-feeding insect UV light traps and sticky traps were mounted at regular intervals on the walls of the facility. The animals were also examined for the presence of ticks.

The five animals with even numbering (2, 4, 6, 8, 10) each received 2 ml of 5 log TCID_50_/ml of the recombinant virus, LSDV Saratov/2017, intravenously (called the infected/inoculated group – IN) and the remaining five animals with odd numbers were mock inoculated (called C1 - in-contact group 1, also acting as negative controls) with phosphate-buffered saline (PBS) (Table 1). The animals were placed in a row along a shared trough according to their consecutive numbering. Their mobility was restricted using tethering, although contact between adjacent animals was possible. At post-inoculation (p.i.) day 33, when there were clear signs of infection in the C1 animals (e.g. crusts, shedding), another group of five bulls (C2 group) was introduced, and positioned between the clinically ill animals, including infected and in-contact animals (Table 2).

**Table 1:** Schematic of animal allocation (“-” – the first group of in-contact animals (C1), “+” – virus inoculated animals).

№ animals									
C1-1	IN-2	C1-3	IN-4	C1-5	IN-6	C1-7	IN-8	C1-9	IN-10
−	+	−	+	−	+	−	+	−	+

**Table 2:** Positioning of the second group of in-contact animals (C2) and clinical signs measurement in all animals.

№ animals														
C1-1	IN-2	C1-3	C2-3	IN-4	C1-5	C2-4	IN-6	C2-1	C1-7	IN-8	C2-5	C1-9	C2-2	IN-10
−/+	−/−	+/+	−/−	+/+	+/+	−/−	+\+	−/−	+/+	−/+	−/−	+/+	−/−	+/+

“−/−” - no clinical signs, “−/+” - mild clinical signs, “+/+” - pronounced clinical signs.

The animals were monitored daily for the presence of fever until day 52 and clinical signs of LSD until the end of the experiment. To evaluate the time course of virus shedding and viremia, nasal and ocular discharges, skin scabs, blood and semen were collected and tested for virus presence using PCR until day 50.

To assess the clinical picture and collect samples, all experimental animals were first stunned using a penetrative captive bolt to induce unconsciousness, followed by the administration of “Adilinum super” (Federal Center for Toxicological, Radiation and Biological Safety, Kazan, Russia) at day 61 p.i. at a recommended dose of 5 mg/kg according to the drug use instruction approved by the Russian Federal Service for Veterinary and Phytosanitary Surveillance in 2008. At the recommended dose, the Adilinum mechanism of action provides painless and rapid euthanasia: cerebral death commences first followed by circulatory collapse.

### Virus isolation in cell culture

Only skin lesions were used for virus isolation and culture. They were ground finely and added to sterile saline at a mass/volume (m/v) ratio of 1:10, followed by repeated freeze–thaw cycles at −80 °C and room temperature (3 times) to disrupt cell membranes. The processed samples were clarified at 1,500 rpm for 15 minutes (min). The supernatant was removed, mixed with antibiotics (penicillin and streptomycin at final concentrations of 2000 IU/mL and 2 mg/mL, respectively) at room temperature for 90 min and then inoculated onto ovine testis or goat gonad cells (70–80% confluency for both), as follows: after removal of the growth medium from flasks containing the cell cultures and washing twice with Hanks’ medium, a 0.1 mL volume of inoculum was added. The flasks were incubated at 37 °C for 90 min to ensure virus adhesion. Once the incubation period was completed, 10 mL of maintenance medium supplemented with 1.0 mL fetal bovine serum was added. The flasks were again incubated at 37 °C. The inoculated cell cultures were observed daily for cytopathic effect (CPE). The cells were then harvested when 80% CPE was observed and lysed to release the virus, using repeated freeze- thaw cycles (3×). The presence of LSDV was confirmed using real-time PCR.

### Virus neutralization

Virus neutralization in flat-bottomed microplates (96 wells) was conducted using the protocol described previously^24^, with a few modifications. The test was performed on ovine testis cells using 2 replicates. The volume of inocumlum virus was 100 µl into each well. The neutralization index was considered negative if less than or equal to1:8.

### ELISA testing

A double-antigen ELISA for the detection of antibodies against capripoxviruses, including lumpy skin disease virus (LSDV), sheeppox virus (SPPV) and goatpox virus (GTPV) in serum or plasma from cattle, sheep, goats or other susceptible species (IDvet, France), was used to confirm exposure of the bulls to LSDV infection. Serum samples were collected from infected (IN) and in-contact animals (C1 and C2) (all animals per group) showing clinical signs at 0, 42 and 60 days p.i. The ELISA was performed according to the manufacturer’s recommendations. Reactions were measured as optical density (OD) readings at 450 nm using a Tecan Sunrise absorbance microplate reader (Switzerland). The antibody responses were represented as sample-to-positive (S/P) ratios (percentages), calculated as follows: S/P% ratio = (sample OD– negative control OD)/(positive control OD– negative control OD) × 100%. S/P ratios ≥ 30% were considered as being positive.

### DNA extraction

The samples were aseptically handled and processed as 10% homogenates in PBS. A 200 μL aliquot was used for total nucleic acid extraction using the QIAamp DNA Mini Kit (Qiagen, Germany), following the manufacturer’s recommendations.

### Real-time PCR (quantitative [q] PCR)

Sample extracts were analyzed using real-time PCR for the presence of LSDV DNA, as previously described by Sprygin *et al*.^25^. The fluorogenic probe was labeled at the 5′ end with the FAM reporter dye and with BHQ as a quencher at the 3′ end. Selected primers and probes were synthesized by Syntol (Moscow, Russia). PCR was performed using a Rotor-Gene Q (Qiagen, Germany) instrument and the following thermal-cycling profile: 95 °C for 10 min, followed by 45 cycles at 95 °C for 15 seconds (s) and 60 °C for 60 s. The final reaction volume was 25 μL containing 10 pmol of each primer, as well as 5 pmol of the probe, 25 mM MgCl_2_, 5 μL 5× PCR Buffer (Promega, USA), 1 μL of 10 pmol dNTPs (Invitrogen, USA), and deionized water to make up the final volume. Samples were tested and results interpreted according to the protocol, as previously described^25^.

## Results

### Clinical observations

#### Body temperature

**Figure 1:**
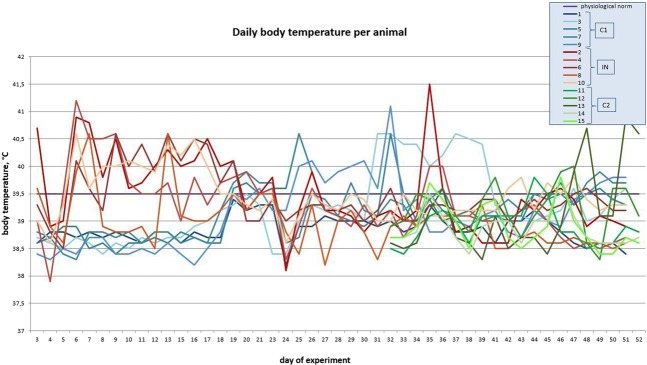
Average body temperature measurements per group over the course of the trial (orange – IN bulls, blue – C1 bulls, grey – C2 bulls).

The baseline average temperature for the animals before the trial was 38.4 °C. All animals infected with LSDV at the outset of the trial (group IN) had fevers up to 40.7 °C (range 39.0 °C to 40.7 °C) on p.i. day 3, indicating virus replication and circulation. By day 8 p.i., the body temperatures of all the inoculated bulls rose above 39.6 °C and their temperatures remained high until p.i. day 20, and thereafter moderate declines were measured to within the range of 38.1 to 39.6 °C in most of the infected animals up to around day 40 p.i. – thereafter a second bout of fever (bi-phasic) was measured in most of the group (except for NI-8) with temperatures rising to a high of 41.5 °C (IN-2) on day 41 p.i., tailoring off to close to normal levels from day 48 p.i. (Fig. 1 and S1 Table).

On the other hand, the control (C1) in-contact animals that did not receive virus at initial inoculation, maintained normal body temperatures of around 38.6 °C until day 19 p.i., and thereafter their temperatures also showed increases up to 41.1 °C (C1-9, day 46 p.i.), which decreased slightly on day 46 p.i., but then rising again (bi-phasic fever) in most animals (except C1-1) up until the end of the trial, i.e., day 61.

In C2 animals, which were introduced from day 33 p.i., their body temperatures increased from as early as five days post-introduction (day 38 of the trial), also showing a bi-phasic pattern of fever, with the second phase starting around day 49 p.i. (16 days post-introduction), with a high of 40.9 °C being recorded for animal C2-3.

#### Edema

**Figure 2:**
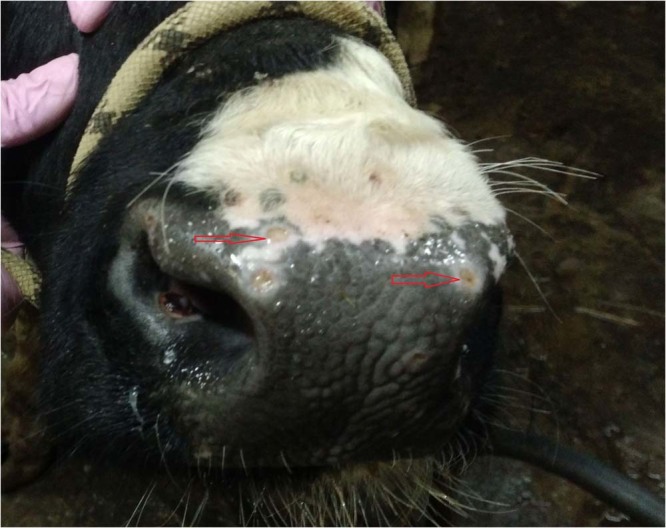
Lesions in the muzzle (red arrows) of IN-6 at day 21 p.i.

**Figure 3:**
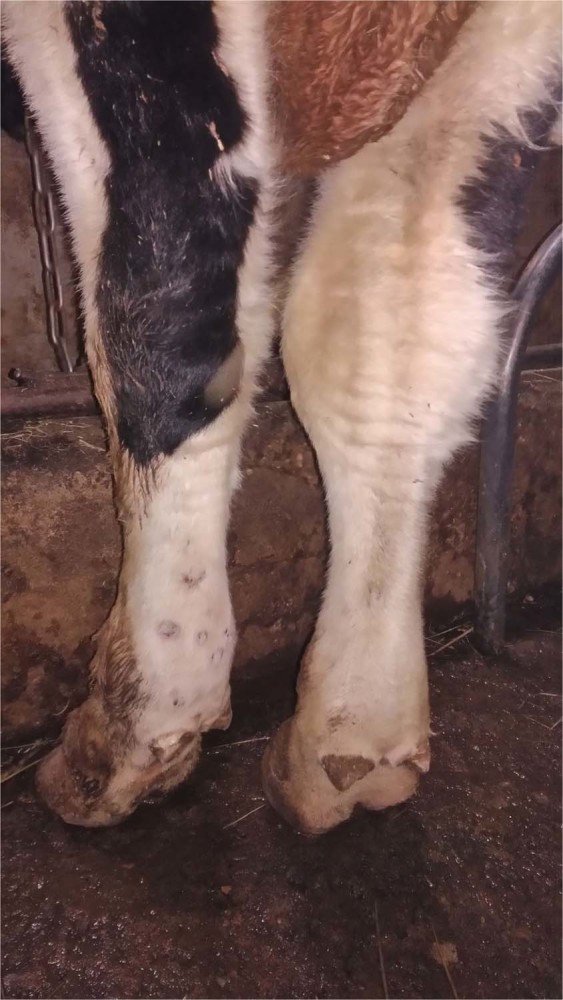
Edematous joints in IN-6 bull at day 21 p.i.

There was a significant level of edema in the inoculated bulls (IN group) in their subscapular axillary and popliteal lymph nodes, except in bull IN-6 – this animal exhibited edema in its dewlap area and joints, accompanied by lesions on its muzzle, and general weakness (Figs. 2 and 3). In the C1 group, edema appeared late, between days 28–30 p.i., in the areas under the jaw, corresponding with the detectable viremia and elevated body temperatures in all the bulls in this group (up to 40.6 °C, in C1-5) (Figs. 5 and 6). In the C2 group, elevated temperatures were recorded in all animals within a week following their inclusion in the trial (day 33 p.i.), found to be associated with slightly enlarged lymph nodes and edema in the jaw (for C2-3), and the C2-1 bull displayed a mild edema in its dewlap.

**Figure 4:**
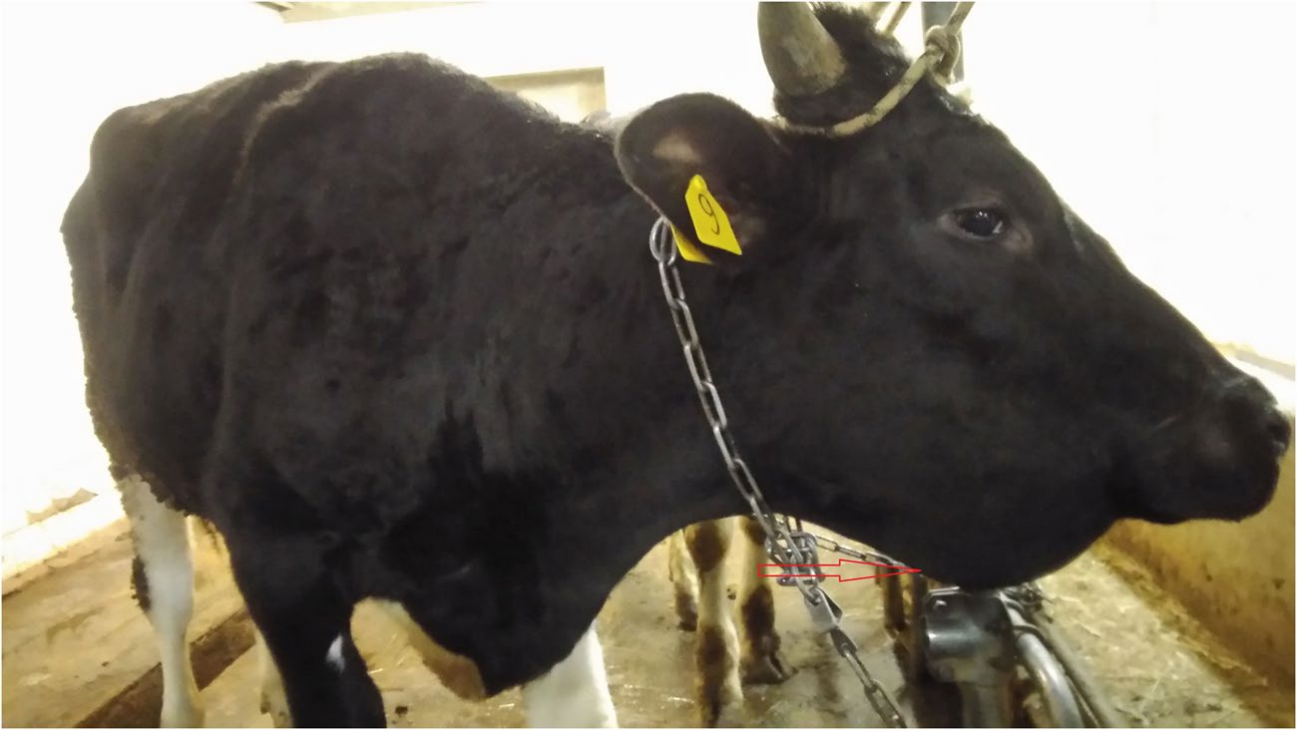
In-contact bull C1-9 with swollen submandibular space (red arrow) at day 28 p.i.

**Figure 5:**
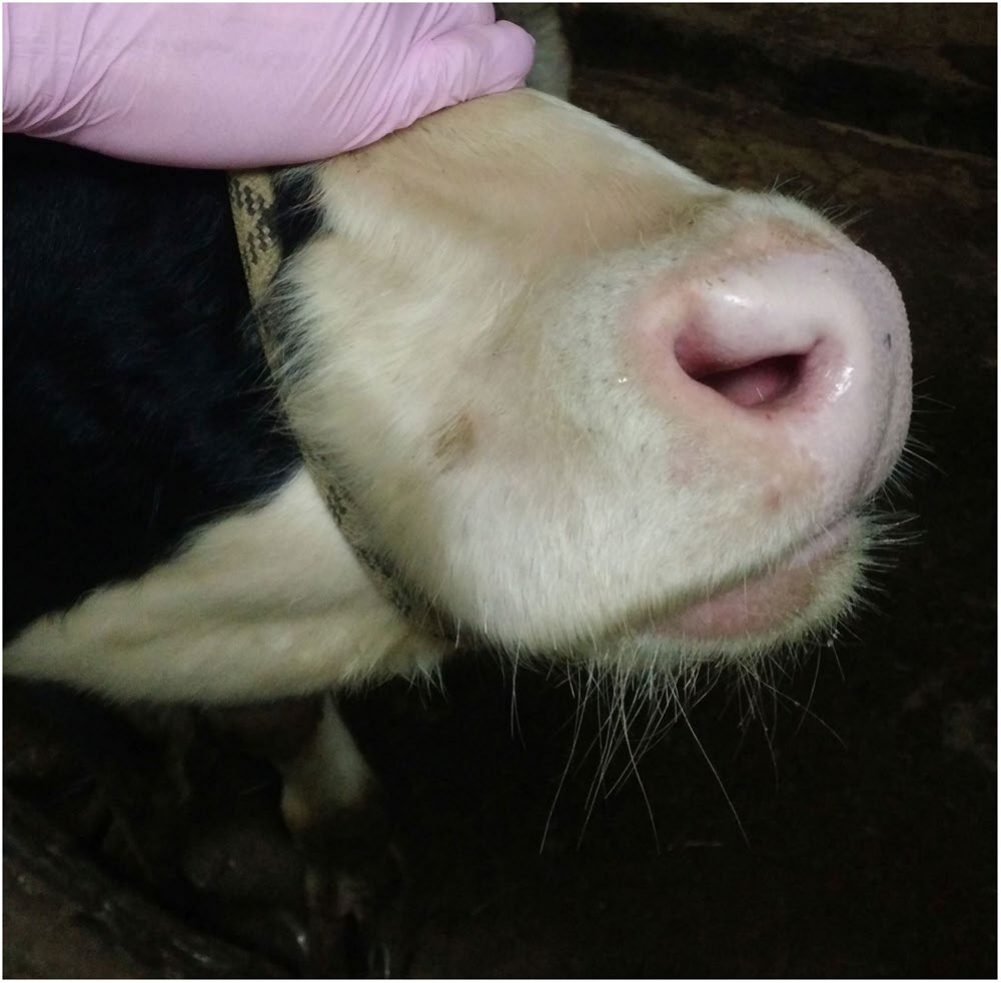
C1-5 bull with hyperaemic muzzle epithelium at day 28 p.i.

**Figure 6:**
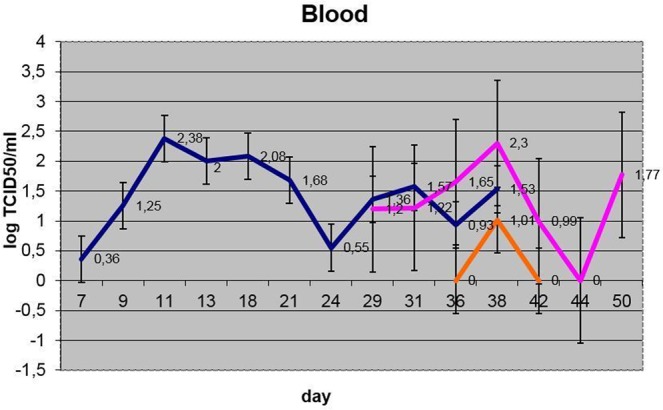
Viremia dynamics (averaged per group) (blue – IN, pink – C1, orange – C2).

#### Viremia

Viremia, as assessed using qPCR on blood samples, was evident as early as p.i. day 7 in the infected animals (IN-6), peaking by p.i. days 11-13 (only detectable on day 13 in IN-8), followed by a slow decline after p.i. day 20, until p.i. day 38 in all animals in this group (with no detection in IN-8, indicating a very low level of viremia in this animal) (Fig. 6, S1 Table).

In the C1 control (in-contact) animals (except in C1-1), which were not inoculated, viremia was measurable using qPCR only after p.i. day 27, to a varying extent, and reached a plateau between p.i. day 38 and 41 (4 out of 5 bulls), which corresponds to days 25–28 following the peak viremia in infected (IN) bulls. Viremia in IN and C1 animals became undetectable after p.i. day 43 (except in C1-3, day 47).

For the C2 group, except for a low-level detectable viremia five days post-introduction in C2-3, no viremia was detectable in blood for the remaining animals, or at any other time point during the trial for this group, even though a low-level bi-phasic fever was measured. However, low levels of viral DNA was detectable from nasal and/or ocular secretions from all animals from this group at some point during their inclusion in the trial (S1 Table).

#### Virus shedding

**Figure 7:**
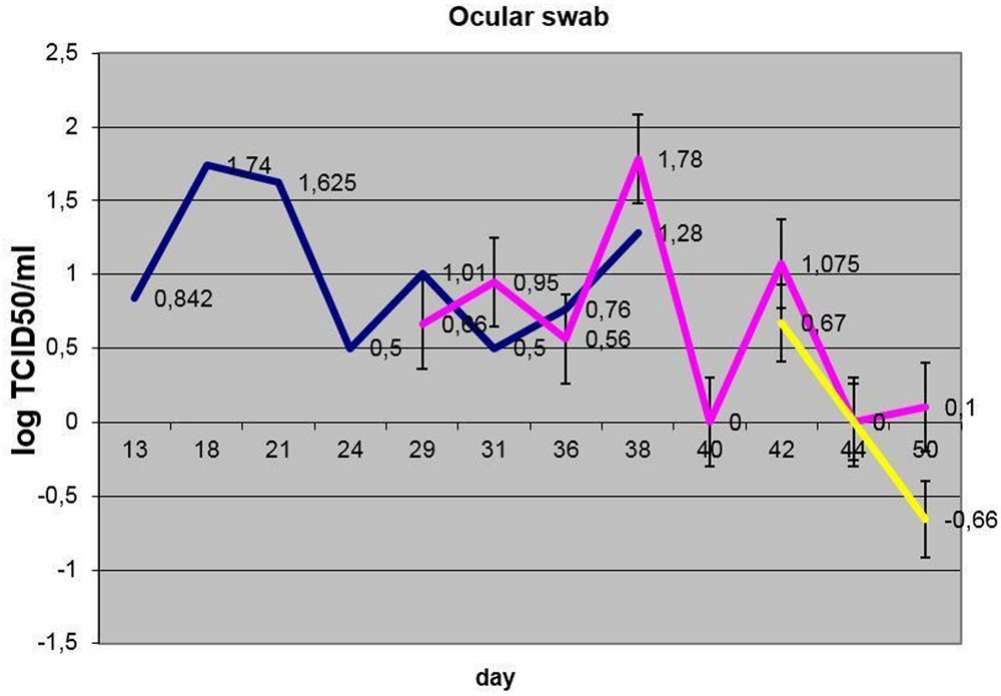
Dynamics of ocular shedding (averaged per group) (blue – IN, pink – C1, yellow – C2).

**Figure 8:**
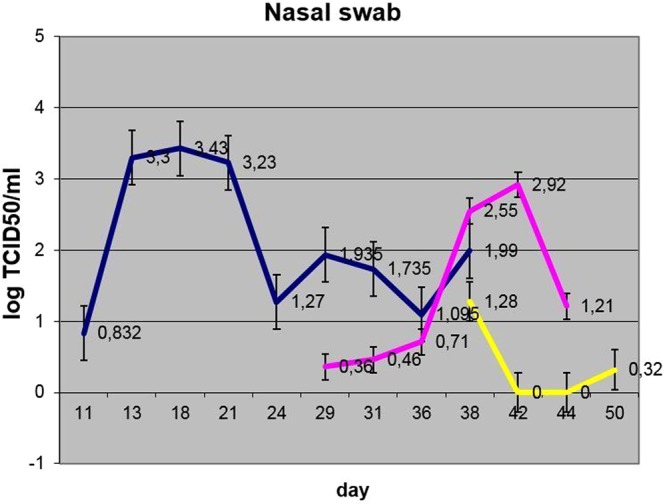
Dynamics of nasal shedding (averaged per group) (blue – IN, pink – C1, yellow – C2).

Virus shedding in inoculated bulls was initially observed as early as p.i. day 11 in nasal discharges, and this lasted intermittently, at least until p.i. day 38. Ocular shedding was also observed in nos. 2, 4, 6 and 10, starting from day 13 p.i. (S1 Table, Fig. 7). Nasal shedding was observed in bull nos. 2, 4, 6 and 10 by day 11 p.i. (S1 Table and Fig. 8).

The dynamics of virus shedding in the two groups in contact with each other at the outset of the trial (IN and C1) showed no statistically significant differences (p > 0.05). The nasal shedding was more durable in IN bulls versus C1 animals with no statistical significance in virus loads. The ocular shedding was comparable in time between the two groups and did not differ statistically (Figs. 7 and 8).

#### Skin lesions

In the inoculated bulls, by p.i. day 11, there was a significant appearance of lesions (0.5 to 1 cm diameter) on the shoulders of three of them (bull nos. 4, 6 and 10). In addition, in these bulls in this group, except IN-2, the scrotum and ventral aspects of the abdomen showed typical foci of erosions. Bull IN-2 showed erosions in only the groin and scapula areas, with the temperature remaining steady at an average of 40 °C. By day 15, there was fulminating disease presentation in infected bull nos. 4, 6 and 10 with symptoms of multiple lumps, coalescing together over the entire body (up to 3 cm in diameter), erosions in the foci up to 2 cm in diameter, and lesions in the scapula and sides as large as 1.5 cm in diameter. Erosions were also observed on their muzzles and associated epithelia. Bull no. 8 displayed similar symptoms, but their appearance was delayed. By p.i. day 21, the size of the lesions had enlarged to 2.5 cm in diameter in inoculated bull nos. 4, 6 and 10, and their nasal epithelia were hyperaemic. Also, there were isolated foci of necrotic lesions in the scrota of these animals.

**Figure 9:**
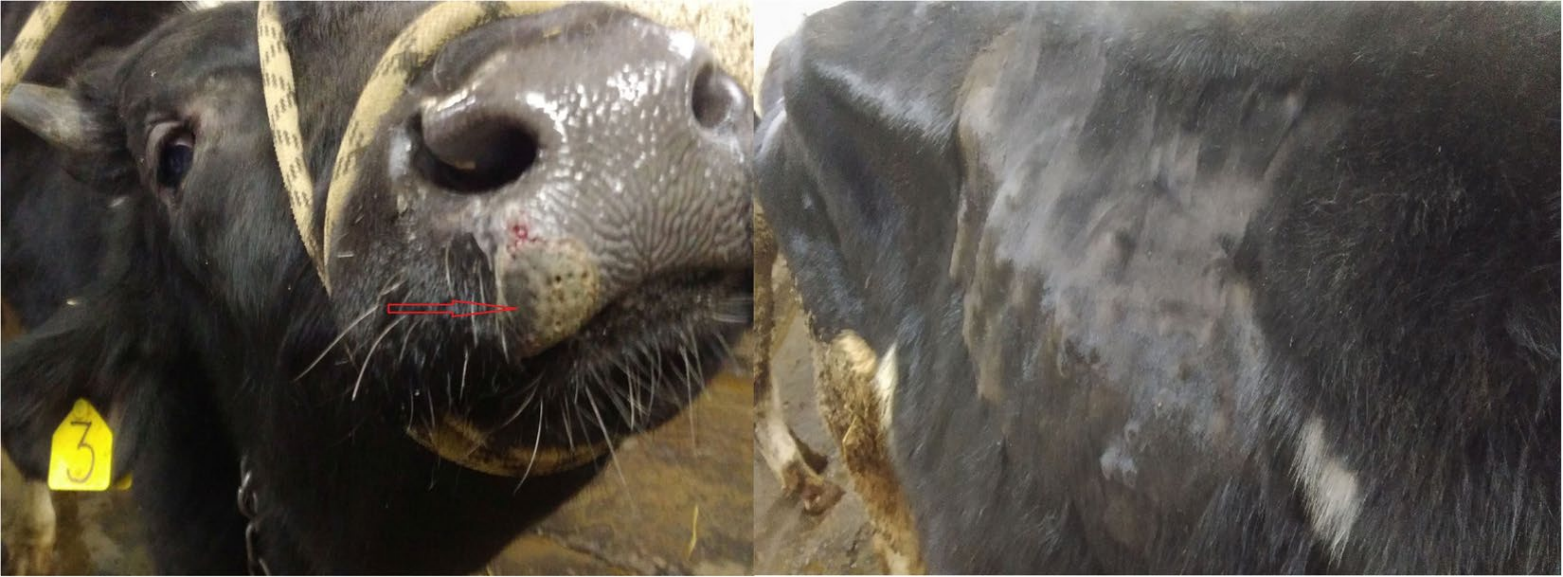
C1 bulls with lesions. (**a**) In-contact bull (C1-3) at day 35 p.i., indicating a lesion in the muzzle (arrow). (**b**) C1-7 bull with skin lumps (lesions) on the back and ventral side.

For the C1 bulls, by day 28 p.i., three of them exhibited small erosions in their nasal epithelia and in their muzzles (Fig. 9a), during which period their viremia was significant, with high body temperatures (40.6 °C in C1-5). Importantly, no erosions were evident towards the rear half of their bodies (scrotum or inner sides of their legs), as opposed to small skin lesions present on their necks. Three out of five C1 animals (C1-5, C1-7, C1-9) developed characteristic signs of viral infection by day 35, with the symptoms including: enlarged lymph nodes (predominantly prescapular, paratracheal and head lymph glands) and multiple lumps over their bodies, from their heads to their tails, 0.5 to −4 cm in diameter (Fig. 9b). However, there was no pronounced pathology in popliteal or groin lymph glands as compared to the inoculated animals, and also there were no erosions in their scrotums.

Blood, nasal and ocular discharges were positive for LSDV DNA from these animals, as assessed using qPCR. Even though bull C1-1 was asymptomatic, it was positive for the presence of virus using qPCR analysis. The presence of virus in ocular shedding became detectable only from p.i. day 28 (nos. 3, 5, 7 and 9), while virus shedding in nasal excretions was evident mostly after 35 days p.i. (S1 Table, Figs. 6–8). Ocular shedding was more pronounced in C1 bulls as compared to the inoculated bulls (IN group), although, in general, the inoculated bulls displayed longer and more intense shedding.

**Figure 10:**
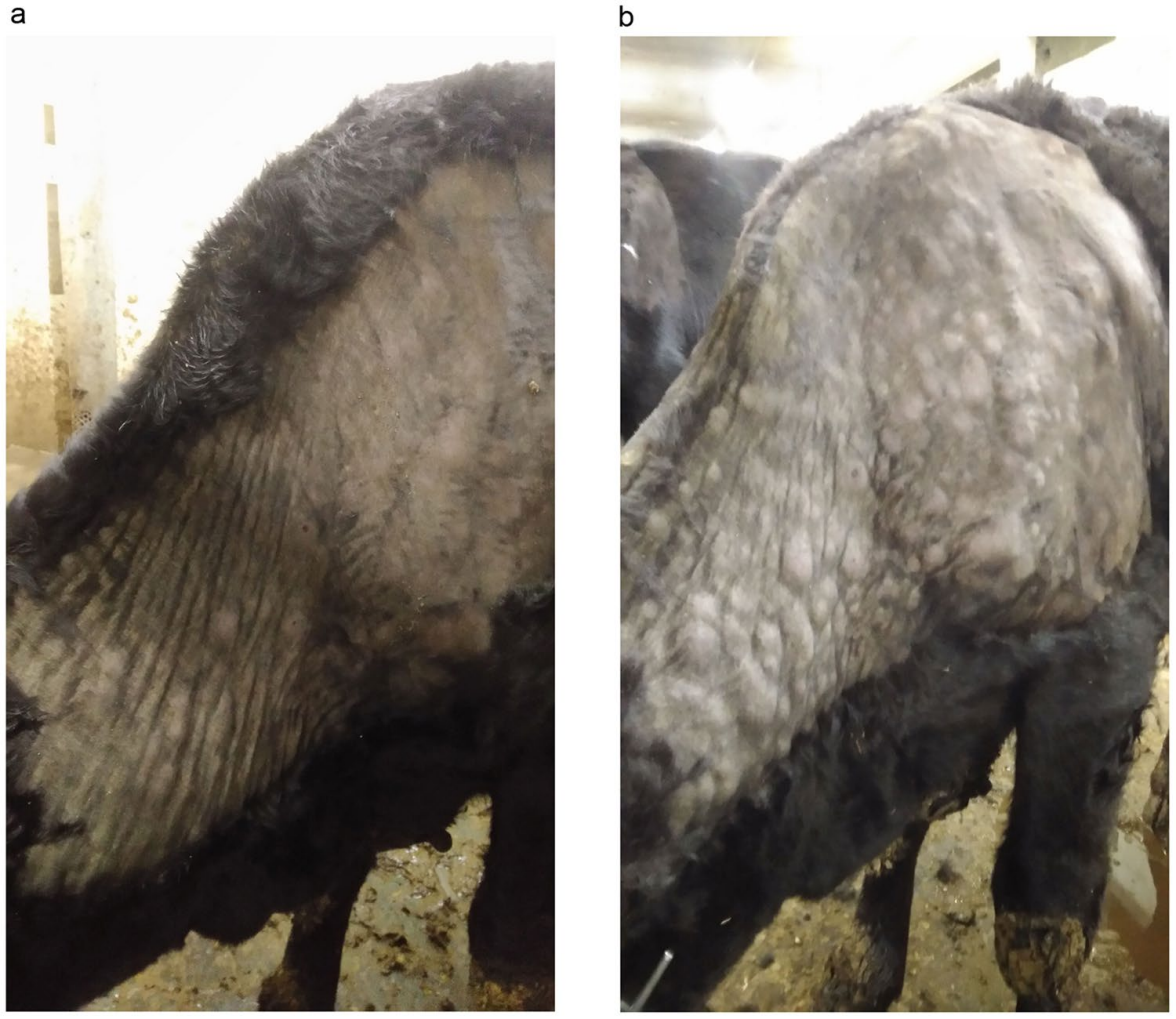
C2-3 bull: (**a**) at day 20 post-introduction - lumps developing on the neck; (**b**) at day 26 post-introduction – lump development progressing towards the tail.

From day 38 of the experiment, i.e., five days after introducing the C2 animals, viral DNA was detectable from them in blood (viremia), ocular and nasal discharges (S1 Table, Figs. 6–8). Shedding in C2 bulls was weak and lasted intermittently for 12 days, from day 5 to day 17 post-introduction. There were variable clinical symptoms in the C2 animals: C2-1 displayed a mild edema in its dewlap, whereas C2-5 developed a few foci of erosion in its scrotum (Table 2). At post-introduction day 21 (i.e., day 54 of the experiment), the C2-3 bull displayed a few small lumps in the scapular region. By post-introduction day 26, these lumps had increased in number and size, between 2.0 and 2.5 cm in diameter (Fig. 10a,b).

#### Recovery from infection

Inoculated (IN) animal nos. 4, 6 and 10 had persistent clinical symptoms, with virus shedding in secretions, till p.i. day 37. But, the inoculated bulls nos. 2 and 8 were asymptomatic, without fever, despite delivering positive PCR results for the presence of virus. All the infected (inoculated) bulls stopped shedding the virus thereafter, with erosions displaying complete scarring and their edemas being cleared, but with their lymph nodes remaining enlarged (as detectable to the touch) up to p.i. day 45.

On the other hand, most of the C1 bulls (nos. 3, 5, 7, 9) displayed clinical signs until day 54. Their skin lesions became necrotic and erupted, with persistent fever between 39 and 40 °C. At p.i. day 50, these bulls developed additional skin lesions, hyperemic nasal epithelia, mild edema in their jaws and dewlaps, and enlarged lymph nodes in their necks. They also had enlarged lymph nodes that were painful to the touch. There was still detectable virus shedding, at this time.

### ELISA and VNT

ELISA testing was conducted to assess the levels of seropositivity of the animals to the virus, and it was confirmed that at day 0 before the inoculation, all animals were seronegative (Table 3). By p.i. day 42, the five IN bulls seroconverted, whereas, only three out of the five in-contact group (C-1) showed a weak seropositive response (Table 3). By p.i. day 60, all IN bulls were strongly seropositive, which was also verified using virus neutralization testing (VNT), as were all C1 animals. However, C2 animals were seronegative throughout the experiment, until day 60, even though there was the detectable presence of virus in their nasal and ocular discharges (and, on one day, day 38, in the blood of bull C2-3).

**Table 3:** ELISA and VN testing of sera from selected animals from the different groups for antibodies to LSDV.

Animal	ELISA At day 0	ELISA At day 42 p.i.	ELISA At day 60 p.i.	VNT at day 60 p.i.
IN 2	negative	105%	151%	1:32
IN 4	negative	110%	198%	1:64
IN 6	negative	155%	200%	1:64
IN 8	negative	78%	132%	1:32
IN 10	negative	105%	190%	1:64
C1-1	negative	55%	135%	1:32
C1-3	negative	47%	109%	1:16
C1-5	negative	negative	118%	1:32
C1-7	negative	negative	160%	1:32
C1-9	negative	41%	106%	1:16
C2-1	negative	negative	negative	<1:8
C2-2	negative	negative	negative	<1:8
C2-3	negative	negative	negative	<1:8
C2-4	negative	negative	negative	<1:8
C2-5	negative	negative	negative	<1:8

## Discussion

Recombination is key towards molecular evolution of viruses. Many viruses appear to have diverged from a common ancestor through genetic exchanges and reassortments to expand their diversity, likely including capripoxviruses^26^. However, novel features arising in new chimeric progeny as a result of these events may include drastic phenotypic changes such as a shift in host range, pathogenicity and in transmission pathways^27^. The first field evidence of capripoxvirus recombination between a virulent and vaccine strain was published recently, with the new virus clustering outside both distinct groups of vaccine and field strains^4^. Despite the genome delineation, the degree to which the genome rearrangements contributed to new phenotypic characteristics remains to be elucidated. In this study, we follow up on the novel recombinant strain, LSDV Saratov/2017, in susceptible hosts (bulls) and demonstrate clearly for the first time non-vector in-contact transmission of this isolate with a focus on the time course of viremia and virus shedding in different groups of experimental animals.

The experiment was designed to gain an insight into whether the novel vaccine-derived virulent recombinant LSDV strain, with some genes restored to the yet unknown wild-type parental strain, such as the KSGP-like LSDV isolate, could exert new features surmised to be absent in the normal field parental strains (e.g. Dagestan/2015^20^) – in this case, efficient non-vector-assisted transmission. Previous experiments with field strains never showed convincing proof that LSDV can transmit to susceptible hosts through air-borne or other non-vector contact modes^20,21^. By contrast, the findings of the current study showed that the recombinant virus was able to transmit horizontally without arthropod assistance, as neither flying insects nor ticks were detected throughout the trial period in the indoor containment facility. The first group of in-contact animals (C1) possibly became infected via indirect contact through sharing food and water troughs, while actual physical contact with adjacent animals to their left and right was also possible and may have resulted in direct contact transmission via the mucosa resulting from virus shedding. Drinking troughs have already been blamed for indirect transmission of LSDV in an insect-free setting^17^, however the complexity of transmission is far from being fully comprehended^11,18^.

Interestingly, the viremia in the IN bulls was observed between 7 and 22 days p.i., up to 38 days p.i., whereas Babiuk *et al*. (2008) previously showed that DNA in blood was detectable between 6 and 15 days p.i., only. In the same study, oral and nasal shedding was reported between 12 and 18 days p.i., whereas in our study this window was from 11 to 38 days p.i., which is much longer.

Importantly, virus loads in blood (viremia), nasal and ocular shedding did not differ significantly between IN and C1 animals (p > 0.05), but the duration of viremia and nasal shedding was twice as long in IN animals versus C1 animals, except for ocular shedding where the duration was comparable (S1 Table). It is likely that the intravenous route, which closely imitates an insect bite, promotes a more aggressive disease pattern as evidenced by experimental studies^20,21^. However, the presumed indirect contact (air-borne or alimentary [mucosa]) route as evidenced by C1 bulls in this study deserves further attention in experimental settings to delineate the complex nature of LSDV transmission. The first evidence of viremia in C1 bulls was observed at day 29 p.i. (S1 Table), which is eight days after the IN animals started showing the last signs of their detectable viremia (day 21 p.i.). To confirm the infection, in support with the PCR results, C1 animals did seroconvert, with three of them (C1-1, C1-3 and C1-9) showing antibodies to LSDV by day 42 p.i. (Table 3, ELISA results), with the remaining animals having all seroconverted by day 60 p.i. (Table 3).

Concerning the C2 group, these animals also became infected via an in-contact mode as evidenced by clinical signs, PCR and virus isolation. Although this group had a significantly lower virus load (viremia) versus the IN and C1 groups (p < 0.05), the nasal and oral discharge loads in this group did not differ significantly from either of the other groups (p > 0.05) (Figs. 6–8). As the number of days and duration of detectable virus was also generally lower, it is surmised that there was possible environmental contamination of the swabs with virus from the IN and/or C1 animals.

Of note is that the ELISA and VNT results indicate that the C2 group did not seroconvert, even after 27 days, possibly due to the shorter time available for antibodies to be elicited in this group compared to the other groups (Table 3), whereas virus shedding was detected within the time frame of the experiment (S1 Table). The presence of the virus in nasal and ocular discharges in these animals can be explained by the profound shedding of the virus from the skin erosions in the IN and C1 groups contributing to a more rapid infection of the C2 group. In this regard, the importance of the crusts and erosions in virus transmission, be it vector-borne or in-contact, may be significant. However, as judged by the virus dynamics in nasal and ocular discharges (Figs. 7 and 8), the virus shedding in terms of titers did not differ statistically (p > 0.05), but, when considering the generalized disease presentation in IN bulls with fulminating skin lesions, containing high concentrations of virus, it is proposed that the skin lesions may have contributed to the virus transmission to the C1 in-contact animals, although to a lesser degree in C2 animals. This may suggest that the amount of virus shed by infected animals is key to virus spread. However, the limitation of the reported evidence is that the mechanism of virus entry cannot be conclusively established on account of the observations made during the experiment - this could be either through the alimentary and/or digestive tracts.

In addition, the localization and dissemination of skin lesions merits further discussion. From our experience, inoculated animals usually develop generalized disease with lesions breaking out evenly over the entire body, including scrotum, muzzle and epithelium, and within the same basic time period. This observation was also true for the IN bulls in this study. The C1 animals, in contrast, displayed a dramatically different pattern, where skin lesions first appeared and were initially restricted to the neck and head regions only, with no lesions in the scrotum or remainder of the body. However, eventually new lesions did develop, spreading throughout the remainder of the animals, down to the tail (Fig. 10a,b). This is an interesting observation which may provide clues as to the gateway for LSDV infection through contact. In this scenario it is likely that the virus entered C1 animals through their lungs or other mucosa from where it travelled to the closest skin areas – head and neck - then spreading from there to the rest of the animal via the lymphatic system. Since upon intravenous inoculation the virus skips the skin barrier (such as is the case for an insect bite), it infects macrophages and settles in secondary sites, such as lymph nodes and testicles, whereas upon proposed inhalation and/or contact with conjunctiva, it needs to make its way through respiratory barriers. It is also likely that if the C1 and C2 animals had been given more time, their antibody responses would have likely been more pronounced. Moreover, C2 animals displayed no viremia, although they were in contact with virus-shedding cattle - this may also argue for the virus load factor in LSDV transmission, wherein a certain threshold needs to be surpassed.

Another important aspect of the current findings begs the question as to which genomic loci of the recombinant virus likely contributed to enhanced severity and improved capacity for contact transmission. Although identification of the particular genetic targets determining the virus phenotype was outside the scope of the current study, a number of the vaccine-derived genes within the recombinant virus, which reverted to the wild-type genotype following recombination, warrant further study^4^. Since this virus clearly showed contact transmission, it follows that all LSDVs should be capable of spreading by the same mode to varying degrees. Along with the anticipated vector-borne mode of transmission, with no single vector species yet clearly identified as being responsible for transmission, the direct or indirect contact modes have far reaching implications regarding control and eradication. The previously pursued intravenous pathway resulting from vector blood-feeding, has failed to explain outbreaks occurring when cold or dry weather conditions do not permit potential vector flight activity^1^. The likely reality is that a number of modes probably come into play, with the dominant one leading to an outbreak dependent on the specific circumstances and environmental and biological factors present immediately prior to the onset of the outbreak. This aspect warrants urgent attention and research efforts in the context of the current low-level research base on LSD, especially in non-endemic countries, to gain better insights to enable more adequate and targeted control practices to be developed and implemented.

Assimilating the evidence gained from this study, a contact mode of LSDV transmission has been clearly demonstrated for a novel recombinant LSDV recently recovered in the field. Coupled to other alternative modes like vector-assisted spread, these findings may contribute to future risk analysis and complement approaches to combating the threat this disease poses. The findings presented herein will lay a justifying basis for revisiting the control strategies currently in place, considering the danger from the emergence of hybrids, should the use of live vaccines be resorted to, especially in non-endemic countries. Further studies into LSDV biology are warranted to delineate the reasonable question if the contact mode demonstrated here is a *de novo*-created feature absent from both parental stains of the novel LSDV or whether it maintained a low profile, but was activated by genetic alterations following recombination.

Overall, the current approach to combat LSD and the virus, generally looks at insect vector control, besides vaccination and restriction of cattle movement, while food, water or litter are often overlooked as being ineffective sources of infection. In this scenario, control strategies need to be revisited for development of a more complex approach involving new parameters to consider in risk analyses. Moreover, contact transmission mitigates the factor of seasonality, which is linked to insect activity and widens the possibilities for spread regardless of the presence of biting insects.

## Supplementary information

Supplementary Information.
