# Supplementary material for: Non-vector-borne transmission of lumpy skin disease virus
Source: Sci Rep. 2020 May 4;10:7436. doi: 10.1038/s41598-020-64029-w (PMC7198617; doi:10.1038/s41598-020-64029-w)
Supplement: Supplementary file 1 — Supplementary Information. [file 41598_2020_64029_MOESM1_ESM.doc]

**Non-vector-borne transmission of lumpy skin disease virus**

Kononov Aleksandr, Byadovskaya Olga, Wallace, David B., Prutnikov Pavel, Pestova Yana, Kononova Svetlana, Nesterov Alexander, Rusaleev Vladimir, Lozovoy Dmitriy, Sprygin Alexander

**S1 Table. Virus dynamics as determined using real time PCR**

| **Group** | **day post infection** | **3** | **7** | **9** | **11** | **13** | **18** | **21** | **24** | **29** | **31** | **36** | **38** | **42** | **44** | **48** | **50** | **52** |
| --- | --- | --- | --- | --- | --- | --- | --- | --- | --- | --- | --- | --- | --- | --- | --- | --- | --- | --- |
| IN viremia | 2 |  | 34.84 | 30.19 | 27.06 | 25.06 | 29.60 | 31.28 | 34.22 |  |  |  |  |  |  |  |  |  |
|  | 4 |  | 36.20 | 32.51 | 31.78 | 28.81 | 29.93 |  |  |  | 30.09 |  |  |  |  |  |  |  |
|  | 6 |  | 34.80 | 31.37 | 27.33 | 25.74 | 25.54 | 27.50 | 31.96 | 30.89 |  | 32.56 | 30.26 |  |  |  |  |  |
|  | 8 |  |  |  |  | 35.86 |  |  |  |  |  |  |  |  |  |  |  |  |
|  | 10 |  | 33.37 | 31.30 | 21.58 | 25.80 | 27.31 | 30.22 | 35.92 |  |  |  |  |  |  |  |  |  |
| C1 viremia | 1 |  |  |  |  |  |  |  |  |  |  |  |  |  |  |  |  |  |
|  | 3 |  |  |  |  |  |  |  |  |  |  | 29.17 | 24.41 |  |  |  | 29.31 |  |
|  | 5 |  |  |  |  |  |  |  |  | 34.74 |  | 31.98 | 28.3 |  |  |  |  |  |
|  | 7 |  |  |  |  |  |  |  |  |  |  | 27.99 | 25.59 | 32.33 |  |  |  |  |
|  | 9 |  |  |  |  |  |  |  |  | 28.28 | 31.43 | 29.92 | 30.68 |  |  |  |  |  |
| C2 viremia | 1 |  |  |  |  |  |  |  |  |  |  |  |  |  |  |  |  |  |
|  | 2 |  |  |  |  |  |  |  |  |  |  |  |  |  |  |  |  |  |
|  | 3 |  |  |  |  |  |  |  |  |  |  |  | 32.56 |  |  |  |  |  |
|  | 4 |  |  |  |  |  |  |  |  |  |  |  |  |  |  |  |  |  |
|  | 5 |  |  |  |  |  |  |  |  |  |  |  |  |  |  |  |  |  |
| IN nasal shedding | 2 |  |  |  | 34.42 | 27.84 |  |  | 39.13 |  |  |  |  |  |  |  |  |  |
|  | 4 |  |  |  | 33.74 | 26.05 |  |  |  |  |  |  |  |  |  |  |  |  |
|  | 6 |  |  |  | 28.78 | 18.62 | 19.54 | 21.35 | 22.78 | 24.95 | 24.57 | 31.37 | 28.46 |  |  |  |  |  |
|  | 8 |  |  |  | 37.86 |  |  |  |  |  |  |  |  |  |  |  |  |  |
|  | 10 |  |  |  | 29.91 | 20.78 | 26.14 | 25.92 | 31.81 | 32.37 | 34.33 | 32.51 |  |  |  |  |  |  |
| C1 viremia nasal shedding | 1 |  |  |  |  |  |  |  |  |  |  | 33.93 |  |  |  |  |  |  |
|  | 3 |  |  |  |  |  |  |  |  |  |  | 29.90 | 26.14 | 24.85 |  |  |  |  |
|  | 5 |  |  |  |  |  |  |  |  |  | 34.39 |  |  |  |  |  |  |  |
|  | 7 |  |  |  |  |  |  |  |  | 34.8 |  | 35.4 | 28.13 |  | 31.48 |  |  |  |
|  | 9 |  |  |  |  |  |  |  |  |  |  | 34.45 | 29.56 |  |  |  |  |  |
| C2 viremia nasal shedding | 1 |  |  |  |  |  |  |  |  |  |  |  | 32.41 |  |  |  |  |  |
|  | 2 |  |  |  |  |  |  |  |  |  |  |  | 31.19 |  |  |  | 36.34 |  |
|  | 3 |  |  |  |  |  |  |  |  |  |  |  |  |  |  |  |  |  |
|  | 4 |  |  |  |  |  |  |  |  |  |  |  | 29.96 |  |  |  | 34.30 |  |
|  | 5 |  |  |  |  |  |  |  |  |  |  |  |  |  |  |  | 34.19 |  |
| IN ocular shedding | 2 |  |  |  |  | 34.12 |  |  | 37.34 |  |  |  |  |  |  |  |  |  |
|  | 4 |  |  |  |  | 34.54 |  |  | 34.56 | 32.18 |  |  | 31.21 |  |  |  |  |  |
|  | 6 |  |  |  |  | 30.86 | 27.49 | 26.92 | 28.21 | 29.92 |  | 32.66 |  |  |  |  |  |  |
|  | 8 |  |  |  |  | 33.38 | 31.64 |  | 36.70 | 34.51 |  | 35.21 |  |  |  |  |  |  |
|  | 10 |  |  |  |  | 31.69 | 29.10 | 32.86 | 34.43 | 34.28 | 34.26 | 31.79 |  |  |  |  |  |  |
| C1 ocular shedding | 1 |  |  |  |  |  |  |  |  |  |  | 35.48 |  |  |  |  | 35.72 |  |
|  | 3 |  |  |  |  |  |  |  |  | 34.00 |  |  |  | 32.13 |  |  |  |  |
|  | 5 |  |  |  |  |  |  |  |  | 31.40 | 33.79 | 35.31 |  |  |  |  |  |  |
|  | 7 |  |  |  |  |  |  |  |  | 34.13 | 31.20 | 32.82 |  |  |  |  | 34.30 |  |
|  | 9 |  |  |  |  |  |  |  |  | 34.89 |  | 32.48 | 29.26 | 31.90 |  |  | 37.30 |  |
| C2 ocular shedding | 1 |  |  |  |  |  |  |  |  |  |  |  |  | 32.63 |  |  | 38.76 |  |
|  | 2 |  |  |  |  |  |  |  |  |  |  |  |  | 33.57 |  |  |  |  |
|  | 3 |  |  |  |  |  |  |  |  |  |  |  |  | 34.64 |  |  |  |  |
|  | 4 |  |  |  |  |  |  |  |  |  |  |  |  |  |  |  |  |  |
|  | 5 |  |  |  |  |  |  |  |  |  |  |  |  |  |  |  |  |  |

Red – IN bulls, green – C1 bulls, yellow – C2 bulls. Grey color – negative PCR results.
